# Evidence for common ancestry and microevolution of passerine-adapted *Salmonella enterica* serovar Typhimurium in the UK and USA

**DOI:** 10.1099/mgen.0.000775

**Published:** 2022-02-23

**Authors:** Yezhi Fu, Jared C. Smith, Nikki W. Shariat, Nkuchia M. M’ikanatha, Edward G. Dudley

**Affiliations:** ^1^​ Department of Food Science, The Pennsylvania State University, University Park, PA 16802, USA; ^2^​ Department of Population Health, Poultry Diagnostic and Research Center, University of Georgia, Athens, GA 30602, USA; ^3^​ Pennsylvania Department of Health, Harrisburg, PA 17120, USA; ^4^​ *E. coli* Reference Center, The Pennsylvania State University, University Park, PA 16802, USA

**Keywords:** *Salmonella *Typhimurium, passerines, larids, pseudogenes, virulence gene signatures

## Abstract

The evolution of *Salmonella enterica* serovar Typhimurium (*S*. Typhimurium) within passerines has resulted in pathoadaptation of this serovar to the avian host in Europe. Recently, we identified an *S*. Typhimurium lineage from passerines in North America. The emergence of passerine-adapted *S*. Typhimurium in Europe and North America raises questions regarding its evolutionary origin. Here, we demonstrated that the UK and US passerine-adapted *S*. Typhimurium shared a common ancestor from *ca*. 1838, and larids played a key role in the clonal expansion by disseminating the common ancestor between North America and Europe. Further, we identified virulence gene signatures common in the passerine- and larid-adapted *S*. Typhimurium, including conserved pseudogenes in fimbrial gene *lpfD* and Type 3 Secretion System (T3SS) effector gene *steC*. However, the UK and US passerine-adapted *S*. Typhimurium also possessed unique virulence gene signatures (i.e. pseudogenes in fimbrial gene *fimC* and T3SS effector genes *sspH2*, *gogB*, *sseJ* and *sseK2*), and the majority of them (38/47) lost a virulence plasmid pSLT that was present in the larid-adapted *S*. Typhimurium. These results provide evidence that passerine-adapted *S*. Typhimurium share a common ancestor with those from larids, and the divergence of passerine- and larid-adapted *S*. Typhimurium might be due to pseudogenization or loss of specific virulence genes.

## Data Summary

Sequence data of the *S*. Typhimurium strains are deposited in the NCBI Sequence Read Archive (https://www.ncbi.nlm.nih.gov/sra). Accession numbers are available in Tables S1 and S2 (available in the online version of this article).

*Salmonella enterica* serovar Typhimurium (*S*. Typhimurium) is considered a generalist with a broad host range; however, certain *S*. Typhimurium variants are associated with specific hosts and appear to be host-adapted. Such variants include the definitive phage types (DT) DT8 and DT46 in ducks [1], DT2 and DT99 adapted to feral pigeons [2], and sequence type (ST) ST313 being responsible for invasive non-typhoidal *Salmonella* disease among humans [3–5]. Previous epidemiological surveys in European countries (e.g. the UK [6, 7], Sweden [8], Norway [9] and Germany [1]) have reported that passerine salmonellosis is caused by specific *S*. Typhimurium phage types, i.e. DT40 and DT56(v). Because these two phage types are frequently identified among passerines but only occasionally isolated from other host species, it is thought that these serovar Typhimurium variants have undergone adaptation within passerines [10].

**Fig. 1.**
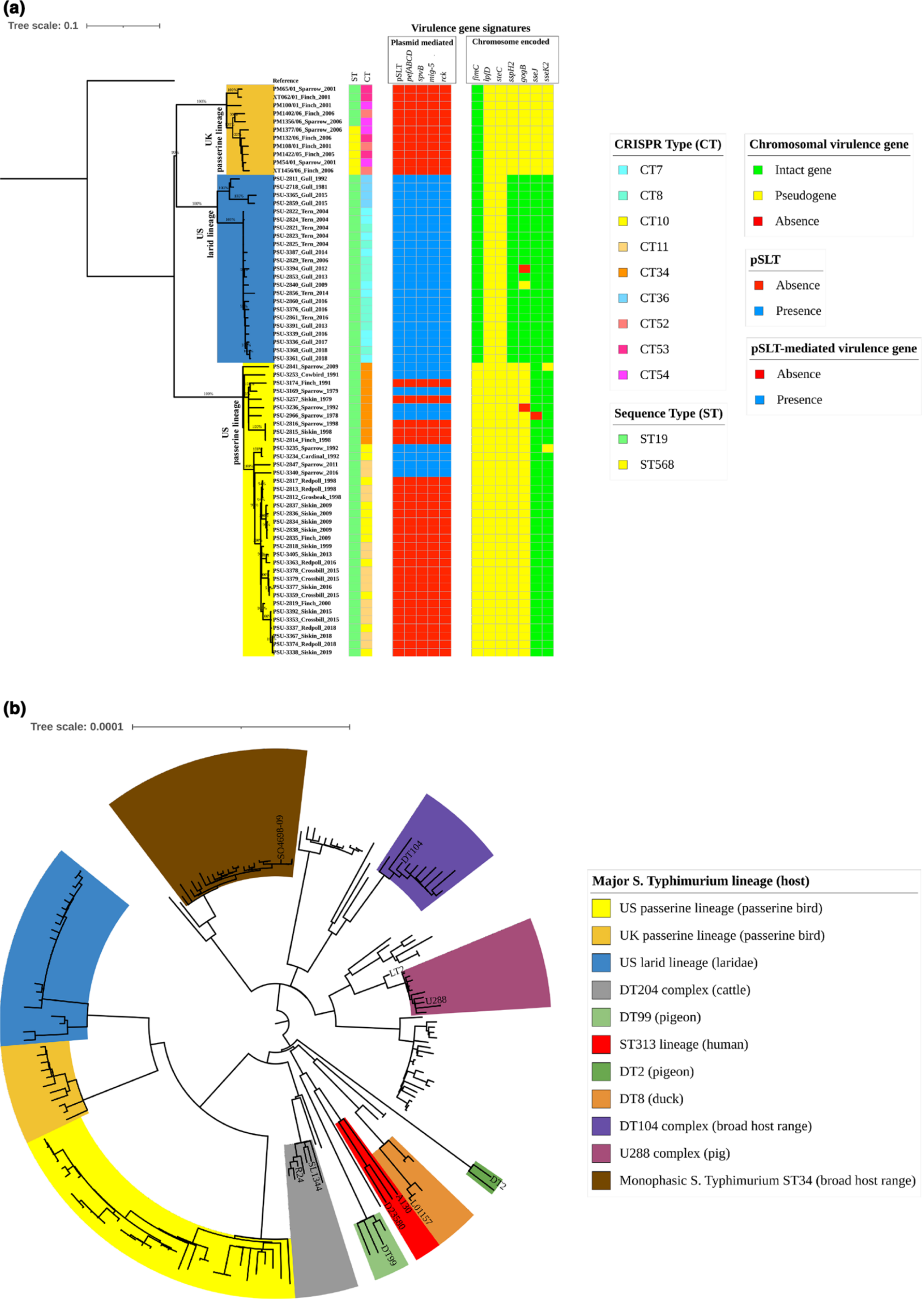
Phylogenetic analysis of the UK and US passerine-adapted *S*. Typhimurium isolates. (**a**) Maximum-likelihood phylogenetic tree of the 70 *S*. Typhimurium isolates from US passerines (*n*=36), US larids (*n*=23) and UK passerines (*n*=11). The tree was reconstructed based on 2943 SNPs in the core genomic regions of the 70 isolates against reference genome *S*. Typhimurium strain LT2 (RefSeq NC_003197.1), reconstructed by mega x (v10.1.8) by using the Tamura–Nei model and 500 bootstrap replicates, and visualized by using iTOL. Three lineages are defined by bird types [i.e. US passerine lineage (yellow), US larid lineage (blue) and UK passerine lineage (orange)]. Bootstrap values are displayed as percentages on the tree branches. The labels at the tree tips represent the isolate name_bird host_isolation year. Colour strips to the right of the tree represent genotypes [sequence type (ST) and CRISPR type (CT)] and virulence gene signatures (see colour key). (**b**) Maximum-likelihood phylogenetic tree of 183 *S*. Typhimurium isolates from various hosts representing the genetic diversity within the serovar based on 11384 SNPs with reference to *S*. Typhimurium LT2. The tree is rooted at the midpoint. Colour ranges in the tree represent the major *S*. Typhimurium lineages identified in the literature. Labels at the tree tips represent the representative isolates from individual lineages and complexes (see colour key). Broad host range in parentheses indicates that isolates from the corresponding lineage are commonly identified among humans, cattle, pigs, poultry, and other hosts or environmental niches. The specific host in parentheses indicates that isolates from the corresponding lineage are primarily from that specific host.

Phylogenetic analysis based on whole-genome sequences has provided an important method to investigate the genetic relatedness of passerine isolates to isolates from other hosts (e.g. humans, livestock or poultry) and identify the genetic changes in serovar Typhimurium that contribute to passerine adaptation. In a previous study in England and Wales, Mather and colleagues sequenced the genomes of 11 isolates from passerine salmonellosis [10]. Single nucleotide polymorphism (SNP) phylogenetic analysis revealed these passerine isolates formed a lineage (henceforth referred to as the UK passerine lineage) distinct from representative isolates from multiple sources, including humans, cattle, horses, chicken and pigeons [10]. Recently, we identified two *S*. Typhimurium lineages (unpublished data) formed by passerine (e.g. sparrows, finches, redpolls, siskins) and larid (i.e. gulls and terns) isolates (henceforth referred to as the US passerine lineage or larid lineage) in a retrospective whole-genome sequencing study on *S. enterica* isolated from wild birds (1978–2019) in the USA. In the present study, to explore the evolutionary history and origin of passerine-adapted *S*. Typhimurium, we reconstructed a maximum-likelihood phylogenetic tree based on the 70 genomes from the US passerine isolates (*n*=36), US larid isolates (*n*=23) and UK passerine isolates (*n*=11) (Table S1), using *S*. Typhimurium strain LT2 (RefSeq NC_003197.1) as the reference genome [11]. We determined that the US larid lineage was more closely clustered with the UK passerine lineage, rather than with the US passerine lineage (Fig. 1a). By adding *S*. Typhimurium representative genomes (isolate names: L01157, DT2, DT99, SL1344, R24, A130, D23580, DT104, SO4698-09 and U288; isolate names are indicated at tree tips in Fig. 1b) [2, 12–18] and context genomes [19] from multiple hosts (Table S2; *n*=114) to the tree, we demonstrated that the three lineages derived from wild birds clustered into a larger clade that was distantly related to the major *S*. Typhimurium lineages identified in the literature [20] (Fig. 1b). The nearest SNP distance between isolates from the three lineages and non-avian isolates was 645.0. Further, the UK passerine isolates were closely clustered (average SNP distance of isolates within the three lineages: 402.4) with the US passerine and larid isolates, indicating potential clonal expansion of the passerine lineage between the USA and the UK. A neighbour-joining (NJ) tree (Fig. S1) of the wild bird isolates (*n*=70) and context isolates from multiple hosts (*n*=114) was built to complement the core genome SNP-based phylogenetic analysis. The NJ tree was reconstructed based on allelic differences in the 21 065 loci of the *Salmonella* whole genome multilocus sequence typing (wgMLST) scheme at EnteroBase. The lineages present in the NJ tree and their genetic relatedness were congruent with those formed in the maximum-likelihood phylogenetic tree based on SNPs (Fig. 1b).

We further classified the bird isolates by two established *Salmonella* subtyping methods, i.e. classic seven-gene MLST [21] and clustered regularly interspaced short palindromic repeats (CRISPR) typing [22], to determine the sequence type (ST) and CRISPR type (CT) of these bird isolates. MLST has become the gold standard for population genetic analyses of pathogenic microbes since its first introduction in 1998 [23]. The classic *Salmonella* MLST technique distinguishes strains within the same serovar according to the nucleotide variations in seven housekeeping genes [21]. However, this subtyping method sometimes lacks discriminatory power due to the sequence conservation in housekeeping genes, limiting its use in evolutionary studies and epidemiological investigations. In the present study, MLST was unable to discriminate the three lineages from each other. MLST indicated that 93 % (65/70) of the bird isolates belonged to ST19, while 7 % (5/70) of the isolates belonged to ST568 (Fig. 1a, Table S1). The five isolates belonging to ST568 were all from the UK passerine lineage. Recent studies suggested that CRISPR typing can provide high resolution for typing of very closely related isolates [24], thus being a powerful alternative to MLST. Generally, the number of repeats/spacers in the CRISPR1 array and CRISPR2 array varies between *S*. Typhimurium strains. Therefore, CRISPR typing can distinguish *S*. Typhimurium strains from various hosts based on the spacer diversity in CRISPRs [22]. In this study, CRISPR typing revealed that the three *S*. Typhimurium lineages contained disparate CTs. Specifically, CT10 (*n*=12), CT11 (*n*=14) and CT34 (*n*=10) were unique to the US passerine lineage; CT7 (*n*=9), CT8 (*n*=10) and CT36 (*n*=4) were exclusive to the US larid lineage; and CT52 (*n*=3), CT53 (*n*=4) and CT54 (*n*=4) only occurred among the UK passerine lineage (Fig. 1a, Table S1). In this instance, CRISPR typing is superior to MLST in differentiating the three lineages.

We identified the genomic signatures associated with antimicrobial resistance and virulence and also profiled plasmid presence in genomes from the bird isolates. All of the bird isolates lacked identifiable antimicrobial resistance (AMR) genes, except the cryptic *aac(6')-Iaa* gene, which is not expressed [25]. The lack of AMR genes was consistent with our previous finding that wild birds in the USA were not a common reservoir for resistant *S*. Typhimurium, possibly because their natural habitats were not heavily contaminated with antibiotics compared to clinical or agricultural settings [26]. Interestingly, all larid isolates (23/23) contained the virulence plasmid pSLT that is commonly present in *S*. Typhimurium [27]; however, none of the UK passerine isolates (0/11) and only 25 % (9/36) of the US passerine isolates harboured this plasmid. Consequently, the pSLT-mediated virulence genes [e.g. *pefABCD* (plasmid-encoded fimbriae), *mig-5* (macrophage-induced gene), *r*ck (resistance to complement killing) and *spvB* ( *Salmonella* plasmid virulence)] were absent in the majority (38/47) of passerine isolates (Fig. 1a). Additionally, conserved pseudogenes were identified in Type 3 Secretion System (T3SS) effectors (*steC*, *sspH2*, *gogB*, *sseJ* and *sseK2*) and fimbrial genes (*lpfD* and *fimC*) when aligning these genes against the corresponding reference genes from *S*. Typhimurium LT2 (Table 1). All the isolates (70/70) from the three lineages had a deletion of GTTTGAGAAT at positions 406–415 of the long polar fimbrial gene *lpfD*, and 97 % (68/70) of the isolates displayed a substitution from A to G at position 147 of the T3SS effector gene *steC*. The presence of the same pseudogenes in the three lineages presents strong evidence that the UK passerine isolates share a common ancestor with the US larid and passerine isolates. In addition to the virulence gene signatures common in the three lineages, the US and UK passerine lineages also possessed unique virulence gene signatures that can differentiate them from the US larid lineage. Specifically, all of the isolates (36/36) from the US passerine lineage had a deletion of C at position 87 of the type 1 fimbrial gene *fimC* and a deletion of G at position 463 of the T3SS effector gene *sspH2* (Table 1); however, all of the isolates (11/11) from the UK passerine lineage harboured pseudogenes attributable to a single base-pair deletion in T3SS effector genes *gogB*, *sseJ* and *sseK2* (Table 1). In contrast, these chromosomally encoded virulence genes were intact in the larid isolates, except that one isolate lacked the *gogB* gene and another isolate had a truncated *gogB* gene (Table 1). The similar signatures shared by the UK and US passerine lineages (i.e. loss of pSLT and its associated virulence genes and pseudogene accumulation in the T3SS effectors) indicate that the two lineages have been undergoing an adaptive microevolution. The pSLT-mediated virulence genes (*pefABCD*, *mig-5*, *ric* and *spvB*), fimbrial genes (*fimC* and *lpfD*) and T3SS effector genes (*steC*, *sspH2*, *gogB*, *sseJ* and *sseK2*) have been demonstrated to contribute to *Salmonella* adhesion, intracellular replication, and virulence in human and mammalian cells [28–34]. Although these genes are required for *S*. Typhimurium virulence among humans or mammals, we speculate that they are dispensable in the passerine host because these genes are predicted to be non-functional as a result of pseudogene accumulation or are totally absent in the bird isolates. Loss of virulence for secondary hosts is a common feature of host adaptation [35]. Comparison of the invasiveness and pathogenicity of larid and passerine isolates among different hosts and cell lines might confirm whether the identified genes play a role in host adaptation.

**Table 1.** Chromosomal virulence gene signatures within the three *S*. Typhimurium lineages from wild birds (yellow: US passerine lineage; blue: US larid lineage; and orange: UK passerine lineage)

Lineage	Chromosomal virulence gene signatures*						
	*lpfD* (length=1087 bp)	*steC* (length=1381 bp)	*fimC* (length=708 bp)	*sspH2* (length=2372 bp)	*gogB* (length=1498 bp)	*sseJ* (length=1234 bp)	*sseK2* (length=1047 bp)
US passerine (*n*=36 isolates)	Deletion of GTTTGAGAAT at position 406–415 (36/36)	Substitution from A to G at position 147 (34/36); insertion of A at position 195 (2/36)	Deletion of C at position 87 (36/36)	Deletion of G at position 463 (36/36)	Substitution from T to C at position 238 (35/36); gene absence (1/36)	Intact gene (35/36); gene absence (1/36)	Intact gene (34/36); substitution at multiple positions (2/36)
US larid (*n*=23 isolates)	Deletion of GTTTGAGAAT at position 406–415 (23/23)	Substitution from A to G at position 147 (23/23); deletion of G at position 1072 (23/23)	Intact gene (23/23)	Intact gene (23/23)	Intact gene (21/23); gene absence (1/23); truncated gene (1/23)	Intact gene (23/23)	Intact gene (23/23)
UK passerine (*n*=11 isolates)	Deletion of GTTTGAGAAT at position 406–415 (11/11)	Substitution from A to G at position 147 (11/11); insertion of A at position 195 (11/11)	Intact gene (11/11)	Substitution from C to A at position 1461 (11/11)	Deletion of T at position 1125 (11/11)	Deletion of G at position 976 (11/11)	Deletion of T at position 494 (11/11)

*The virulence gene signatures are identified by aligning draft genomes from wild bird isolates against the reference genes from *S.* Typhimurium LT2.

**Fig. 2.**
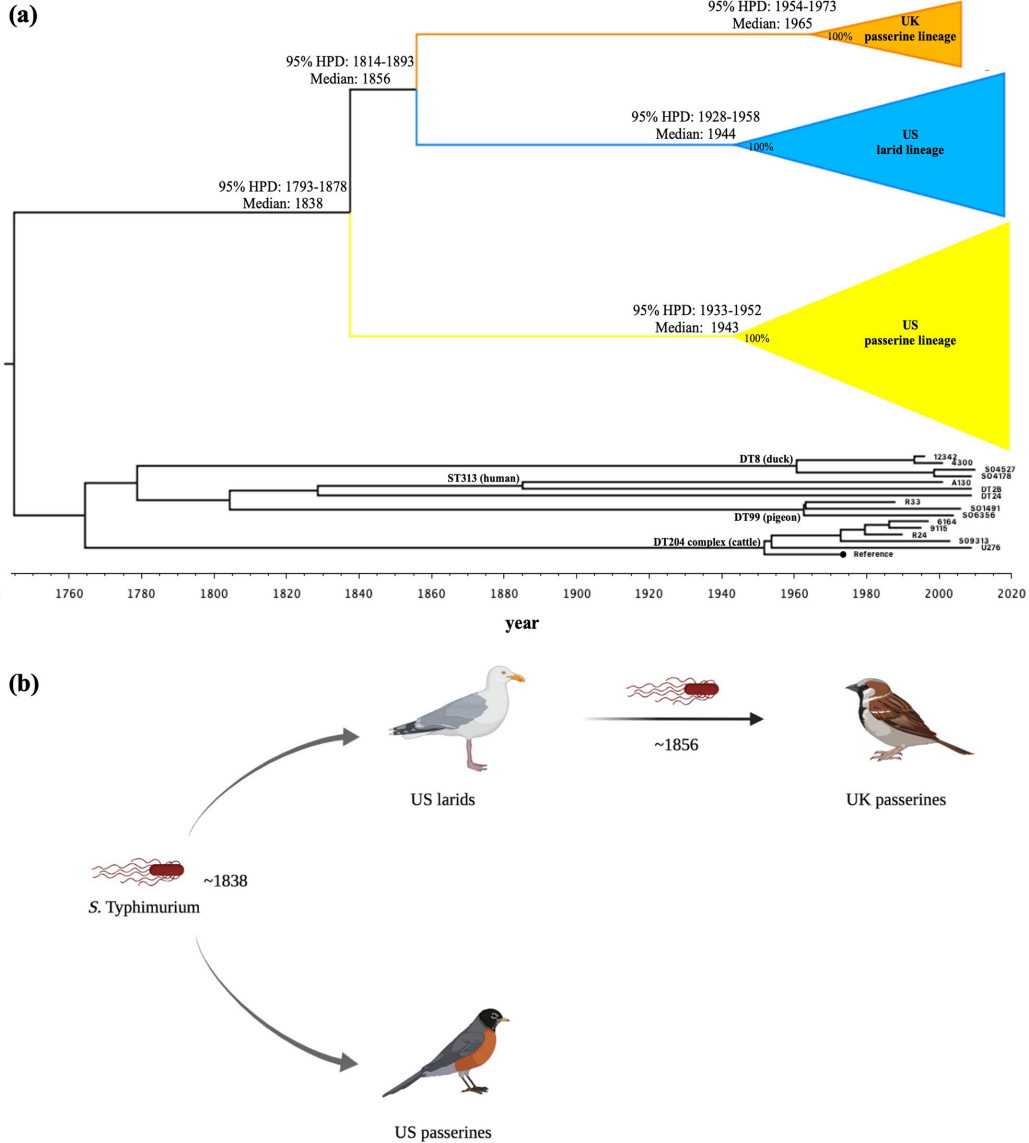
Bayesian phylogenetic inference of the *S.* Typhimurium lineages from wild birds and the potential role of larids (gulls and terns) in the formation of UK passerine-adapted *S.* Typhimurium. (**a**) Time-scaled Bayesian phylogenetic tree of 85 *S.* Typhimurium isolates from US passerines (*n*=36), US larids (*n*=23), UK passerines (*n*=11) and other hosts (*n*=15). The tree was reconstructed by BEAUti (v2.6.5) and BEAST2 (v2.6.5) based on 4347 SNPs in the core genomic regions of the 85 isolates against reference genome *S.* Typhimurium strain SL1344 (NC_016810.1), and visualized by using FigTree (v1.4.4). Median years or range of years on the tree branches represent the 95 % highest posterior probability density (HPD) for the times of the most recent common ancestor for the US passerine lineage (yellow), the US larid lineage (blue) and the UK passerine lineage (orange). Posterior probability values of divergent events are displayed as percentages on the tree nodes. The black circle at the tree tip represents the reference strain SL1344. *S.* Typhimurium lineages (i.e. DT204 complex, ST313, DT8, DT99) formed by other hosts are labelled on the branches. (**b**) Schematic showing the role of the US larids (gulls and terns) in the evolution of host adaptation of *S.* Typhimurium to the UK passerines.

Considering the close phylogenetic relationship among the three lineages examined in this study, we applied a temporal reconstruction using BEAST2 [36] to infer the putative origin of the passerine-adapted *S.* Typhimurium. The formation of the three *S.* Typhimurium lineages is a relatively recent event that has occurred at approximately the same time. As displayed in Fig. 2a), the most recent common ancestor (MRCA) of the three *S.* Typhimurium lineages is estimated to be from *ca.* 1838 [95 % highest probability density (HPD): 1793–1878]. The MRCA evolved among different bird hosts for approximately 100 years and formed the three lineages in this study. Specifically, the US passerine lineage formed in *ca.* 1943 (95 % HPD: 1933–1952); the US larid lineage emerged in *ca.* 1944 (95 % HPD: 1928–1958); and the UK passerine lineage arose in *ca.* 1965 (95 % HPD: 1954–1973). The US larid lineage and the UK passerine lineage had an MRCA and split from each other in *ca.* 1856 (95 % HPD: 1814–1893). On the basis of the Bayesian phylogenetic inference and that the US passerine and larid lineages shared the same genetic signatures (i.e. *lpfD* and *steC*) with the UK passerine lineage (Table 1), the passerine birds in the UK are likely to acquire the ancestral *S.* Typhimurium from the US larids. It is not unusual to observe American herring gulls (a larid) in Europe. In November 1937, Gross observed the first American herring gull off the Spanish coast [37]. Since then, American herring gulls have been recorded in European countries such as Ireland, the UK, France and Norway on a number of occasions [38]. The long-distance migratory capability of American herring gulls and their records in Europe support our hypothesis that larids play a key role in carrying and spreading the ancestral *S.* Typhimurium from North America to Europe. As indicated in Fig. 2b), the US passerines and larids were likely to obtain the MRCA in *ca.* 1838. In *ca.* 1856, the US larids then carried and spread the MRCA to the UK passerine birds. The MRCA further evolved in the UK and the USA toward a passerine-adapted lifestyle. It is worth noting that the schematic illustrated in Fig. 2(b) is hypothetical; the ancestral *S.* Typhimurium was also possibly spread by larids from the UK to the USA, considering that the span of divergence times among the three lineages was less than 20 years (Fig. 2a). A potential *S.* Typhimurium transmission network created at StrainHub supported the latter hypothesis that the common ancestor of the three lineages may have originated from Europe, possibly from cattle (Fig. S2).

In summary, our evidence supports that the passerine-adapted *S.* Typhimurium in the USA share a common ancestor with those from the UK passerine birds, and larids may have played a key role in the formation of the UK passerine lineage by carrying and spreading the ancestral *S.* Typhimurium from the USA to the UK. Further, both the UK and the US passerine-adapted *S.* Typhimurium share similar genetic signatures as defined by the loss of virulence plasmid pSLT and pseudogene accumulation in fimbriae and T3SS effector genes. Our study demonstrates that genome degradation or gene loss might contribute to the early stage of host adaptation [39, 40] of *S.* Typhimurium to passerines. Within-host evolution of serovar Typhimurium is notable because it provides a new opportunity for studying host adaptation. Traditionally, host adaptation of *S. enterica* is studied by genome comparison of broad-host-range serovars such as *S.* Typhimurium to narrow-host-range serovars such as *Salmonella enterica* subsp. *enterica* serovar Typhi [41]. However, the approach is challenging because of the relatively high genetic divergence between two different *S. enterica* serovars [42]. The emergence of host-adapted *S.* Typhimurium pathovariants allows us to identify genes involved in host adaptation by directly comparing the genomes from the same serovar that only differs in host range. However, additional wet-laboratory experiments are needed to confirm the functions of identified genes in pathoadaptation of *S.* Typhimurium to the avian hosts.

**Impact Statement**

Passerine-adapted *Salmonella enterica* serovar Typhimurium (*S*. Typhimurium) have caused salmonellosis in humans and wild birds worldwide. Understanding the emergence, genetic relationship and evolution of passerine-adapted *S*. Typhimurium from different countries is important to better understand *S*. Typhimurium epidemiology, genetic diversity and host adaptation. In the present study, we compare whole-genome sequences of passerine-adapted *S*. Typhimurium from the UK and the USA. Phylogenetic analysis and Bayesian inference suggest that passerine-adapted *S*. Typhimurium from both countries have emerged in recent decades, shared a common ancestor that may be spread by gulls and terns, and formed lineages distinct from the major *S*. Typhimurium lineages originating from humans and domestic animals. Further comparative genomic analysis reveals that the UK and US passerine-adapted *S*. Typhimurium have undergone microevolution to accumulate pseudogenes in specific virulence genes. Our study highlights the importance of wild birds, especially passerines, as carriers of emerging *S*. Typhimurium pathovariants. Additionally, our work advances our understanding of the evolution of *S*. Typhimurium within wild birds. The emergence of passerine-adapted *S*. Typhimurium pathovariants provides us with the opportunity to study the mechanisms that shape the host specificity of *S*. Typhimurium.

## Methods

### Isolate selection

*S.* Typhimurium isolates (*n*=59) from the USA were isolated from dead passerines and larids submitted to the United States Geological Survey – National Wildlife Health Center as part of wildlife mortality and morbidity investigations. The 59 isolates were collected in 21 US states between 1978 and 2019 and sequenced at Penn State University. *S.* Typhimurium isolates (*n*=11) from the UK were isolated from dead passerines through pathological investigations on mortality and morbidity in wild birds across Great Britain conducted at the Institute of Zoology [10]. The 11 isolates were collected in 11 UK counties between 2001 and 2006 and their genomes were publicly available at EnteroBase. The 70 isolates (Table S1) selected were derived from wild birds with confirmed salmonellosis over broad temporal and spatial scales, and had closely genetic relatedness with human clinical isolates [10, 43]. Therefore, the isolates may represent the main *S.* Typhimurium pathovariants emerging in the UK and USA that caused salmonellosis both in humans and in wild birds (i.e. passerines and larids).

### Quality assessment for raw reads

The quality of Illumina paired-end reads of the US isolates sequenced in this study (mean length of the reads: 250 bp) and publicly available UK isolates (mean length of the reads: 100 bp) was assessed using the MicroRunQC workflow in GalaxyTrakr v2 [44]. Raw reads passing quality control thresholds (i.e. average coverage >30, mean quality score >30, number of contigs <400, total assembly length 4.4–5 .l Mb) were used for genomic analysis in this study.

### Phylogenetic analysis

The genetic relatedness of the 70 wild bird isolates (Table S1) was inferred from phylogeny of their core genomes. Snippy (Galaxy v4.5.0) (https://github.com/tseemann/snippy) was used to generate a whole genome alignment and find SNPs between the reference genome LT2 (RefSeq NC_003197.1) and the genomes of wild bird isolates. Snippy-core (Galaxy v4.5.0) (https://github.com/tseemann/snippy) was used to convert the Snippy outputs (i.e. whole genome alignment) into a core genome alignment. The resultant core genome alignment (2943 SNPs in the core genomic regions) was used to reconstruct a maximum-likelihood phylogenetic tree by mega x (v10.1.8) [45] using the Tamura-Nei model and 500 bootstrap replicates. We also generated a maximum-likelihood phylogenetic tree of the 70 wild bird isolates (Table S1) and 114 context isolates (Table S2) from diverse hosts to represent the genetic diversity within serovar Typhimurium. The tree was created using the EnteroBase SNP project with reference to *S.* Typhimurium LT2 [46]. The two SNP phylogenetic trees were visualized and annotated using the Interactive Tree of Life (iTOL v6; https://itol.embl.de). To complement the core genome SNP-based phylogenetic analysis, we built an NJ tree of the 184 isolates from wild birds and other multiple hosts using the *Salmonella* wgMLST scheme (21065 loci) in EnteroBase [46].

### Bayesian inference

A time-scaled Bayesian phylogenetic tree was reconstructed to determine the evolutionary history of the *S.* Typhimurium lineages from wild birds. Before phylogenetic molecular clock analysis, a core genome alignment (4737 SNPs in the core genomic regions) of wild bird isolates (*n*=70; Table S1) and context isolates (*n*=15; Table S2, isolates highlighted in bold) was generated as described above, using the *S.* Typhimurium SL1344 genome (NC_016810.1) as the reference. A root-to-tip plot showing regression of genetic distance against sampling time (Fig. S3) was generated by TempEst [47] to examine the temporal signal of the sequence data, and a previously described date-randomization test was also conducted to confirm the significance of the temporal signal [48]. The parameters for reconstructing the Bayesian phylogenetic tree were set in BEAUti (v2.6.5) [36] as follows: prior assumption-coalescent Bayesian skyline; clock model-relaxed clock log normal with the default clock rate value of 1.0; and Markov chain Monte Carlo (MCMC) at chain length-100 million, storing every 1000 generations. Two independent runs with the same parameters were performed in BEAST2 (v2.6.5) [36] to ensure convergence. The resultant log files were viewed in Tracer (v1.7.2) to check if the effective sample sizes of all parameters was more than 200 and the MCMC chains were convergent. A maximum clade credibility tree was created using TreeAnnotator (v2.6.4) [36] with a burn-in percentage of 10 % and node option of median height. Finally, the tree was visualized using FigTree v1.4.4 (https://github.com/rambaut/figtree/releases). The phylogenetic tree generated for Bayesian inference was also used as an input at StrainHub [49] to build a transmission network of *S.* Typhimurium.

### CRISPR and multilocus sequence typing

CRISPR arrays were identified using CRISPRviz [50]. Spacers were aligned and unique arrays were given a unique allelic identifier as described by Shariat *et al.* [51]. *S.* Typhimurium CRISPR type (CT) was then determined by the unique combination of CRISPR1 and CRISPR2 alleles. In addition, the sequence type (ST) of these *S.* Typhimurium isolates was identified using seven-gene (*aroC*, *dnaN*, *hemD*, *hisD*, *purE*, *sucA* and *thrA*) MLST at EnteroBase [46]. CTs and STs were annotated in the SNP phylogenetic tree.

### AMR, virulence and plasmid profiling

Raw reads of each isolate were *de novo* assembled using Shovill with Trimmomatic (Galaxy v1.0.4) [52]. To identify the antimicrobial resistance genes present in the isolates, each draft genome assembly was searched against the ResFinder database and CARD database using ResFinder 4.1 [53] and Resistance Gene Identifier (v5.2.0) [54]. AMR genes that passed the default threshold values for ResFinder (≥90 % nucleotide identity and ≥60 % coverage) and Resistance Gene Identifier (≥95 % nucleotide identity) were considered to be present in the isolate. Virulence factors were predicted by aligning the draft genome assembly for each isolate against the Virulence Factor database using VFanalyzer [55]. Virulence factors that passed the default threshold values for VFanalyzer (≥90 % amino acid identity and ≥50 % coverage) were considered to be present in the isolate. Below this threshold, genes are defined by ‘absence’, but they may have deletions, insertions or substitutions of interest. We then manually checked the mutation type by aligning the virulence gene of interest against the reference virulence gene from *S.* Typhimurium LT2 using blast (https://blast.ncbi.nlm.nih.gov/Blast.cgi). Plasmid replicon sequences were identified by comparing the draft genome assembly against the PlasmidFinder database using PlasmidFinder 2.1 with search settings of ≥80 % nucleotide identity and ≥60 % coverage [56].

## Supplementary Data

Supplementary material 1
