# Supplementary material for: Evidence for common ancestry and microevolution of passerine-adapted Salmonella enterica serovar Typhimurium in the UK and USA
Source: Microb Genom. 2022 Feb 23;8(2):000775. doi: 10.1099/mgen.0.000775 (PMC8942035; doi:10.1099/mgen.0.000775)
Supplement: Supplementary material 1 [file mgen-8-0775-s001.pdf]

Evidence for common ancestry and microevolution of passerine-adapted *Salmonella enterica* serovar Typhimurium in the United Kingdom and United States

Yezhi Fu, Jared C. Smith, Nikki W. Shariat, Nkuchia M. M'ikanatha and Edward G. Dudley

Supplementary Table S1 Metadata of the *S.* Typhimurium isolates from wild birds (*n* = 70) used in this study (yellow: US passerine isolates; blue: US larid isolates; and orange: UK passerine isolates)

Isolate name	Accesion number	Isolation year	Isolation location	Isolation source	CRISPR type	Sequence type
PSU-2817	SRR12700718	1998	USA:ME	Redpoll	10	19
PSU-2834	SRR12700685	2009	USA:MD	Siskin, Pine	10	19
PSU-2835	SRR12927697	2009	USA:WV	Finch, Purple	10	19
PSU-2836	SRR12700674	2009	USA:WV	Siskin, Pine	10	19
PSU-2837	SRR12700669	2009	USA:VT	Siskin, Pine	10	19
PSU-2838	SRR12927696	2009	USA:WV	Siskin, Pine	10	19
PSU-3234	SRR12927672	1992	USA:DE	Cardinal	10	19
PSU-3235	SRR13061787	1992	USA:DE	Sparrow, House	10	19
PSU-3337	SRR13208234	2018	USA:WI	Redpoll	10	19
PSU-3338	SRR13208223	2019	USA:WI	Siskin, Pine	10	19
PSU-3359	SRR13208220	2015	USA:MT	Crossbill, Red	10	19
PSU-3363	SRR13208216	2016	USA:WI	Redpoll	10	19
PSU-2812	SRR12583006	1998	USA:NH	Grosbeak, Evening	11	19
PSU-2813	SRR12583005	1998	USA:VT	Redpoll	11	19
PSU-2818	SRR12583000	1999	USA:UT	Siskin, Pine	11	19
PSU-2819	SRR12582999	2000	USA:WV	Finch, Gold	11	19
PSU-2847	SRR12700712	2011	USA:NJ	Sparrow, House	11	19
PSU-3340	SRR13208206	2016	USA:MN	Sparrow, House	11	19
PSU-3353	SRR13208226	2015	USA:MT	Crossbill, Red	11	19
PSU-3367	SRR13208211	2018	USA:WI	Siskin, Pine	11	19
PSU-3374	SRR13209069	2018	USA:WI	Redpoll	11	19
PSU-3377	SRR13209053	2016	USA:WI	Siskin, Pine	11	19
PSU-3378	SRR13209052	2015	USA:MT	Crossbill, Red	11	19
PSU-3379	SRR13209051	2015	USA:MT	Crossbill, Red	11	19
PSU-3392	SRR13209070	2015	USA:WA	Siskin, Pine	11	19
PSU-3405	SRR13209057	2013	USA:WI	Siskin, Pine	11	19
PSU-2814	SRR12583004	1998	USA:OH	Goldfinch, American	34	19
PSU-2815	SRR12583003	1998	USA:OH	Siskin, Pine	34	19
PSU-2816	SRR12583002	1998	USA:OH	Sparrow, White-throated	34	19
PSU-2841	SRR12700666	2009	USA:NJ	Sparrow, House	34	19
PSU-2966	SRR12700692	1978	USA:NM	Sparrow, House	34	19
PSU-3169	SRR13061811	1979	USA:WI	Sparrow, House	34	19
PSU-3174	SRR12743058	1991	USA:MO	Goldfinch	34	19
PSU-3236	SRR12927671	1992	USA:KS	Sparrow, House	34	19
PSU-3253	SRR12927723	1991	USA:WA	Cowbird, Brown-headed	34	19
PSU-3257	SRR12927718	1979	USA:WI	Siskin, Pine	34	19
PSU-2822	SRR12582996	2004	USA:MA	Tern, Common	7	19
PSU-2823	SRR12700707	2004	USA:MA	Tern, Common	7	19
PSU-2824	SRR12700696	2004	USA:MA	Tern, Common	7	19
PSU-2840	SRR12700667	2009	USA:ME	Gull, Herring	7	19
PSU-2856	SRR12700702	2014	USA:MA	Tern, Common	7	19
PSU-3336	SRR13208235	2017	USA:ND	Gull, Ring-billed	7	19
PSU-3339	SRR13208212	2016	USA:ND	Gull, Ring-billed	7	19
PSU-3361	SRR13208218	2018	USA:ND	Gull, Franklins	7	19
PSU-3387	SRR13209075	2014	USA:MN	Gull, Ring-billed	7	19
PSU-2821	SRR12582997	2004	USA:MA	Tern, Common	8	19
PSU-2825	SRR12582995	2004	USA:MA	Tern, Common	8	19
PSU-2829	SRR12582991	2006	USA:MA	Tern, Common	8	19
PSU-2853	SRR12700705	2013	USA:DE	Gull, Laughing	8	19
PSU-2860	SRR12700698	2016	USA:ME	Gull, Herring	8	19
PSU-2861	SRR13061812	2016	USA:MA	Tern, Common	8	19
PSU-3368	SRR13208210	2018	USA:MS	Gull, Laughing	8	19
PSU-3376	SRR13209054	2016	USA:ND	Gull, Franklins	8	19
PSU-3391	SRR13209071	2013	USA:MT	Gull, California	8	19
PSU-3394	SRR13209067	2012	USA:SD	Gull, Ring-billed	8	19
PSU-2718	SRR12582963	1981	USA:NY	Gull, Herring	36	19
PSU-2811	SRR13061813	1992	USA:MD	Gull, Laughing	36	19
PSU-2859	SRR12700699	2015	USA:MA	Gull, Herring	36	19
PSU-3365	SRR13208214	2015	USA:ND	Gull, Ring-billed	36	19
PM1402/06	ERS217356	2006	UK:Cheshire	Greenfinch	52	19
XT1456/06	ERS217357	2006	UK:Gwent	Goldfinch	52	568
PM108/01	ERS217358	2001	UK:Powys	Greenfinch	52	568
PM1422/05	ERS217359	2005	UK:Glamorgan	Greenfinch	53	568
PM65/01	ERS217360	2001	UK:Lancashire	House sparrow	53	19
PM132/06	ERS217361	2006	UK:Leicestershire	Greenfinch	53	568
XT062/01	ERS217362	2001	UK:Cheshire	Greenfinch	53	19
PM1377/06	ERS217363	2006	UK:Kent	House sparrow	54	568
PM100/01	ERS217364	2001	UK:Shropshire	Greenfinch	54	19
PM54/01	ERS217365	2001	UK:Nottinghamshire	House sparrow	54	568
PM1356/06	ERS217366	2006	UK:Devon	House sparrow	54	19

Supplementary Table S2 Metadata of the representative and context *S.* Typhimurium isolates (*n* =114) from different hosts used in this study. The isolates highlighted in bold are context isolates used in Bayesian inference

Isolate name	Isolation year	Isolation source	Accession number	Isolate name	Isolation year	Isolation source	Accession number
43397	2014	human	SRR1960212	DT104	NA	NA	HF937208.1
73095	2014	human	SRR1965532	DT12	2009	human	ERR024387
107264	2015	human	SRR3285419	DT120	2009	human	ERR024391
207563	2016	human	SRR3285281	DT135	2009	human	ERR024392
01_2888	NA	pigeon	ERR028072	DT177	2008	human	ERR024394
0309C	2012	pig	SRR6943503	DT193	2009	human	ERR024396
1008-1995	1995	cattle	ERR028312	DT195	2009	human	ERR024397
1013-1997	1995	pig	ERR038760	DT2	NA	pigeon	HG326213.1
10177-1993	1993	pig	ERR038757	**DT24**	2009	human	ERR024388
10246-1993	1993	cattle	ERR038758	**DT2B**	2009	human	ERR024398
10258-1997	1997	turkey	ERR029225	DT7	2009	human	ERR024404
10382-1995	1995	cattle	ERR038759	L01157 (DT8)	NA	duck	ERR744232
10584-1997	1997	NA	ERR024629	DT97	2008	human	ERR024406
10902-1996	1996	environment	ERR028284	DT99	NA	pigeon	ERR019424
10984-1996	1996	cattle	ERR028292	L00041-09	2009	dog	ERR024954
11020-1996	1996	pig	ERR028308	L00961-04	2004	pig	ERR039367
11671-1996	1996	cattle	ERR038761	L01730-06	2006	pig	ERR039368
12005-1995	1995	pig	ERR028290	LT2	1948	NA	NC_003197.1
**12342-1996**	1996	duck	ERR029229	**R24 (DT204)**	1990	cattle	ERR4303294
1402-2000	1995	cattle	ERR028313	**R33**	1988	pigeon	ERR4303379
1446A	2012	pig	SRR6943483	S00250-07	2007	pig	ERR039364
1713-1998	1998	cattle	ERR028294	S00914-05	2005	cattle	ERR028288
1731-1999	1999	pig	ERR038771	S01569-10	2010	pig	ERS023498
1758A	2012	pig	SRR7352631	S02724-05	2005	pig	ERR024634
1939A	2012	pig	SRR6943475	S02909-08	2008	pig	ERR039369
1948B	2012	feed	SRR6943451	S03512-08	2008	cat	ERR038763
2036-2000	2000	feed	ERR029228	S04199-08	2008	pig	ERR029232
2278A	2013	feed	SRR7352633	**S04527-10**	2010	duck	SRR11892970
2798-2001	2001	pig	ERR038774	S04696-09	2009	bird	ERR028277
3193-1995	NA	NA	ERR028272	S04782-03	2003	pig	ERR029235
3203-1997	1997	pig	ERR028291	S05738-08	2008	NA	ERR028282
3543-2002	2002	pig	ERR038775	S05894-09	2009	pig	ERR039371
388-1998	1998	dog	ERR028634	S05968-02	2002	pig	ERR029233
4061-1997	1997	pig	ERR038762	S06221_07	2007	NA	ERR028304
4284-1995	1998	pig	ERR028300	S07292-07	2007	pig	ERR029234
**4300-2001**	2001	duck	ERR029230	S08300-02	2002	pig	ERR028299
4582-1995	1995	cattle	ERR028289	**S09313-03**	2003	cattle	ERR029220
5102-1999	1999	pig	ERR039367	S4489-10	2010	other	ERR038792
547-2001	2001	pig	ERR028283	S5828-08	2008	chicken	ERR038789
5544-1997	1997	environment	ERR028307	**SL1344**	1974	cattle	NC_016810.1
**6164-1997**	1997	chicken	ERR029219	**SO1491-06**	2006	pigeon	ERR028638
6353-1997	1997	pig	ERR028310	SO1960-05	2005	pig	ERR029231
6887-2000	2000	pig	ERR038773	SO3188-03	2003	pig	ERR028314
6940-1998	1998	dog	ERR028303	SO3433-05	2005	chicken	ERR029213
7302-1999	1999	pig	ERR028287	**SO4178-09**	2009	duck	ERR028640
7396-1998	1998	bird	ERR028633	SO4454-08	2008	pig	ERR028636
7828-1995	1995	pig	ERR028285	SO4698-09 (ST34)	2009	NA	GCF_001540845
7830-1995	1995	pig	ERR028286	SO4744-08	2008	chicken	ERR028632
8380-1996	1996	pig	ERR028316	SO5416-06	2006	chicken	ERR029214
8721-1997	1997	environment	ERR028311	**SO6356-04**	2004	feed	ERR028637
8767-1998	1998	pig	ERR028641	SO8313-02	2002	pig	ERR028635
**9115-1996**	1996	cattle	ERR028631	SO9207-07	2007	pig	ERR028315
**A130 (ST313)**	2001	human	ERR023781	SR11-WT	NA	NA	ERR029226
CO119	NA	NA	ERR028643	**U276**	2009	human	ERR024409
CO95	NA	NA	ERR028656	U288	NA	pig	GCF_000380325
D23580 (ST313)	2004	human	GCF_900538085	U302	2009	human	ERR024401
DT1	2009	human	ERR024408	U310	2009	human	ERR024402

Supplementary Figure S1

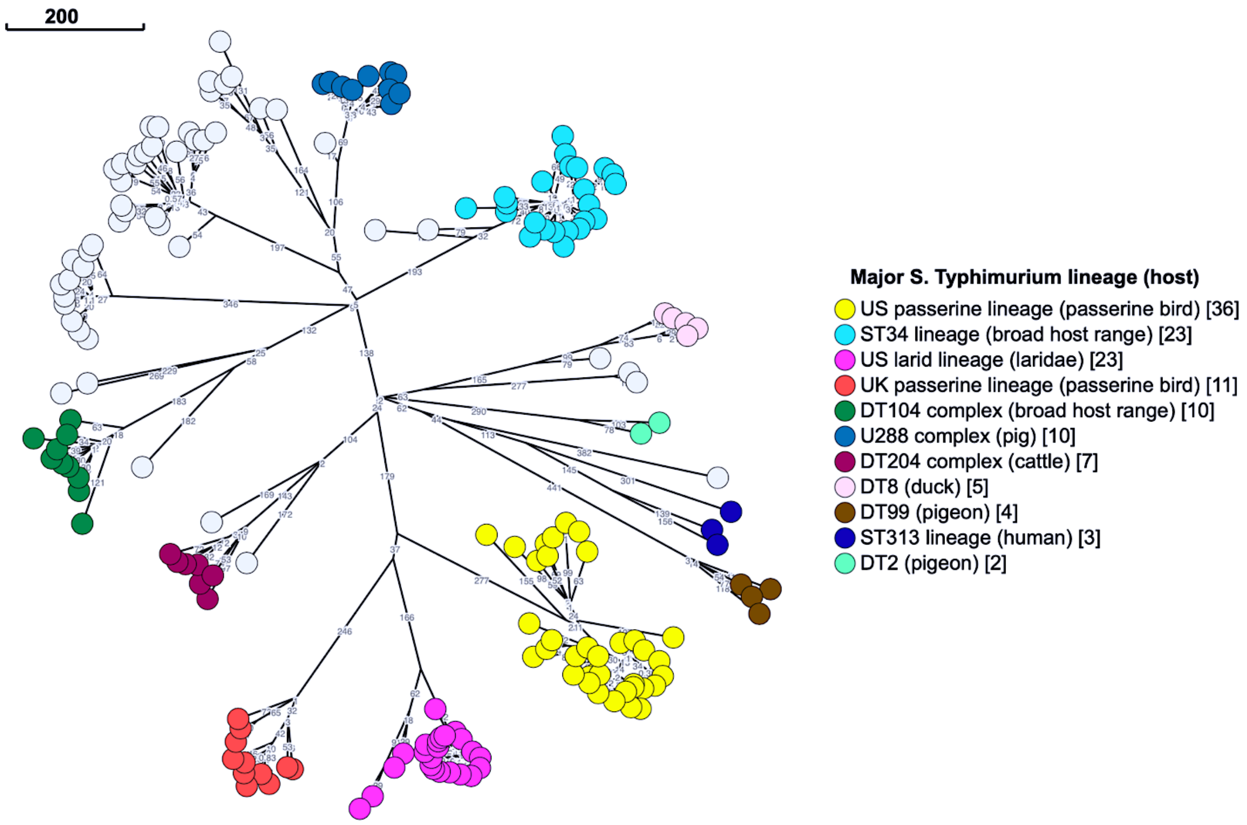

Fig. S1. Neighbor joining tree based on the whole genome multilocus sequence typing (wgMLST; 21065 loci) of the 184 *S.* Typhimurium isolates from different hosts. Tree tips are colored by *S.* Typhimurium lineages (see key), with host and number of isolates listed in round and square brackets in the key. The scale bar indicates 200 wgMLST alleles. Allele differences between isolates are indicated by numbers on the connecting lines.

Supplementary Figure S2

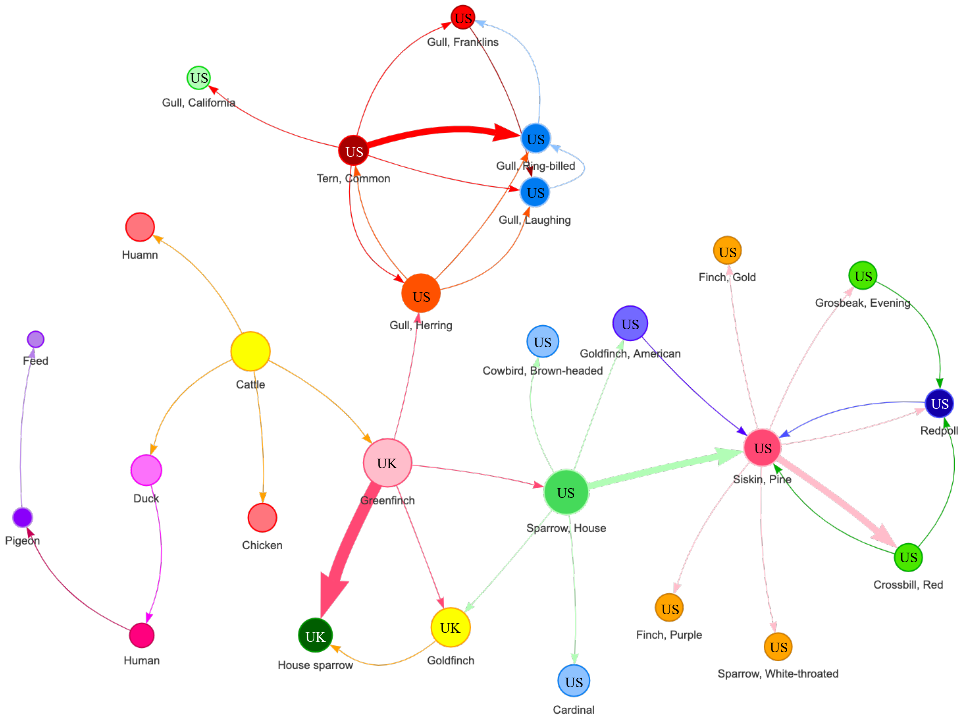

Fig. S2. Potential *S.* Typhimurium transmission network based on hosts. The transmission network is built at StrainHub (http://strainhub.centralus.azurecontainer.io/) by mapping the metadata onto a phylogenetic tree of 85 *S.* Typhimurium isolates from US passerines ($n = 36$), US larids ($n = 23$), UK passerines ($n = 11$), and other hosts ($n = 15$). A parsimony ancestral reconstruction step is performed to create links between the tree and its associated metadata. The nodes represent *S.* Typhimurium isolates. Hosts and geographic locations of the isolates are labeled at the node sides and centers, respectively. Sizes of nodes are scaled by the metric "closeness centrality". Closeness evaluates a node based on the relative sum of the lengths of all the shortest paths from that node to all other nodes within network. The arrows represent transition of *S.* Typhimurium from one host to another host. The thickness of the lines and arrows reflect the transition frequency.

Supplementary Figure S3

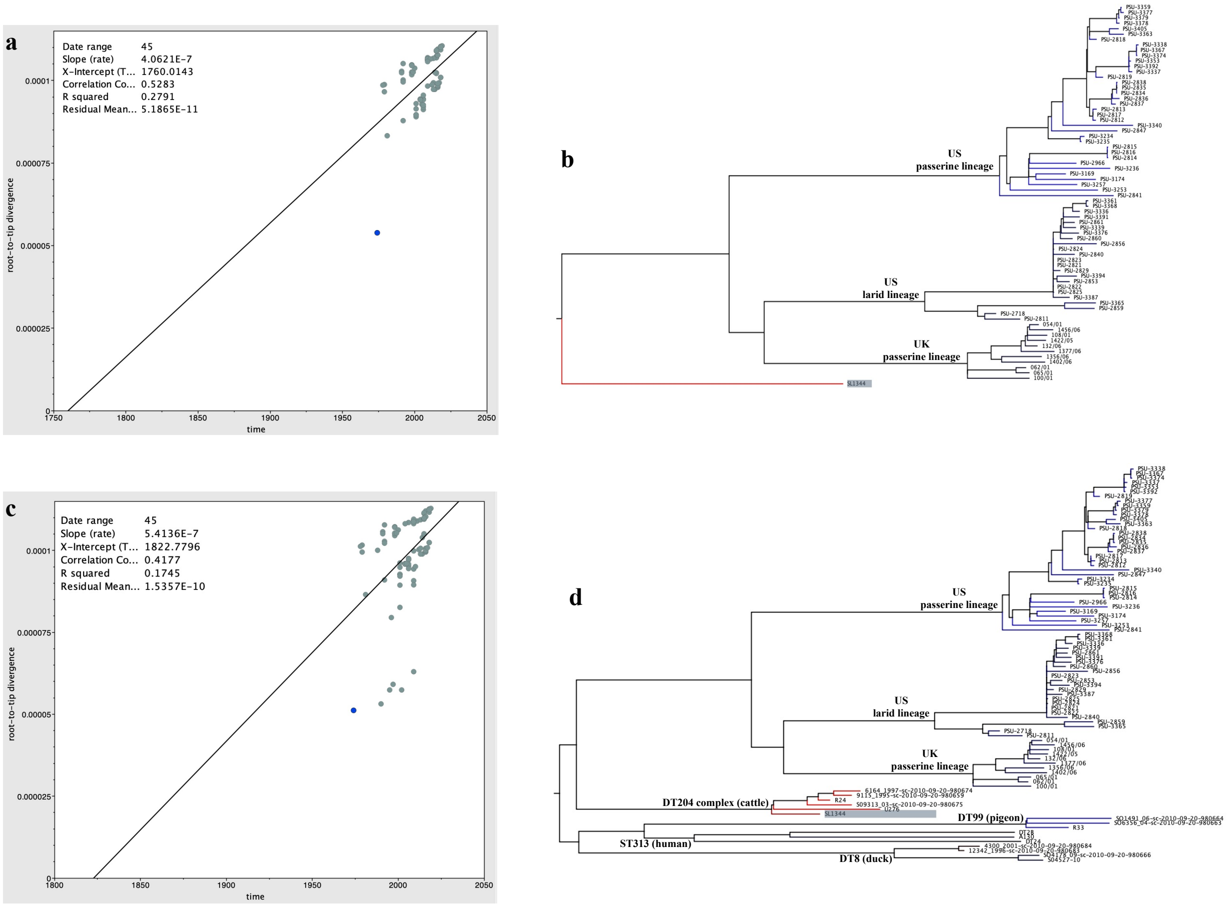

Fig. S3. Temporal signal of the genome sequences used for Bayesian inference. (**a**) Root-to-tip regression plot and (**b**) Phylogeny of 70 *S.* Typhimurium genome sequences from wild birds. (**c**) Root-to-tip regression plot and (**d**) Phylogeny of 85 *S.* Typhimurium genome sequences from wild birds (n = 70) and other hosts (n = 15). Major *S.* Typhimurium lineages and their primary hosts are indicated on the phylogenetic tree branches. Reference genome from *S.* Typhimurium SL1344 is highlighted in blue in (**a**) and (**c**), and shaded in grey in (**b**) and (**d**).
